# Molecular Design in Dynamic (Meth)acrylic Cross-linkers for Tough, Self-healing, and Recyclable Elastomer

**DOI:** 10.1021/acs.macromol.5c00649

**Published:** 2025-07-16

**Authors:** Hoang Nam Nguyen, Li-Hung Lu, Kai Ou, Doan Van Hong Thien, Chun-Jen Huang

**Affiliations:** † Faculty of Automation Engineering, 95400 Can Tho University, 3/2 Street, Ninh Kieu District, Can Tho City 900000, Vietnam; ‡ Department of Chemical & Materials Engineering, 34911 National Central University, Jhong-Li, Taoyuan 32023, Taiwan; § Chung Yuan Christian University, 200 Chung Pei Rd., Chung-Li City 32023, Taiwan; ∥ School of Materials Science and Engineering, The University of New South Wales, Sydney, New SouthWales 2052, Australia; ▲ Faculty of Chemical Engineering, Can Tho University, 3/2 Street, Ninh Kieu District, Can Tho City 900000, Vietnam

## Abstract

The development of high-performance elastomers with customizable mechanical, self-healing, and degradative properties is essential for their utilization in advanced applications and environmental preservation. In this study, three dynamic covalent cross-linkers were synthesized and integrated into acrylic elastomer formulations. Their chemical design explored the effects of aromatic disulfide, α-methyl, urea, and urethane moieties on the elastomers’ physicochemical properties. The findings highlight the versatility of these cross-linkers, demonstrating their critical role in enhancing thermal stability, mechanical performance, self-healing, and recyclability. This research underscores the transformative potential of molecular cross-linker design in advancing recyclable and repairable materials for diverse applications.

## Graphical abstract

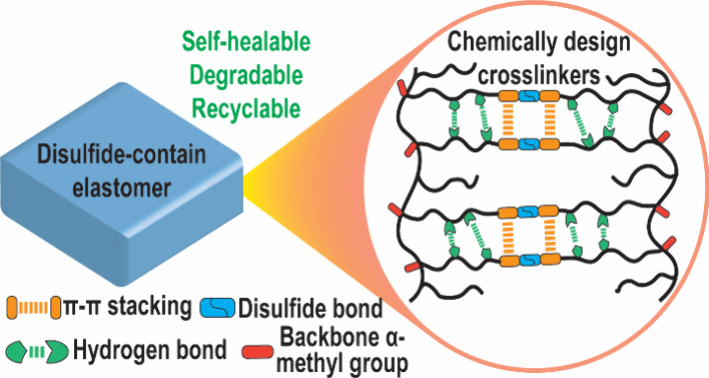

## Introduction

The design and development of universal dynamic cross-linkers have attracted significant attention in recent years due to their potential for creating materials with enhanced mechanical properties, self-healing capabilities, and recyclability.^1^ These features are particularly beneficial in applications where durability and sustainability are paramount.^2^ Despite the progress in synthesizing self-healing elastomers, many existing systems face challenges, including limited mechanical strength, poor healing efficiency under mild conditions, and compromised stability under harsh environments.^3^ To overcome the challenge, intrinsically self-healing materials have been engineered utilizing supramolecular polymer chemistry, facilitating their autonomous reconstruction via dynamic intermolecular interactions, such as hydrogen bonds or metal–ligand interactions.^4–6^ However, most self-healable and recyclable polymeric materials are polyurethane-related polymers but not acrylate-based polymers, which will limit future usage of acrylate-based materials. Therefore, designing universal dynamic cross-linkers that can be applied to various acrylate monomers is crucial and requires a thorough understanding of how chemical structures influence the physical properties and dynamic behavior of the material.

Incorporating reversible covalent bonds, such as disulfide linkages, has been identified as an effective strategy to achieve self-healing properties in elastomers.^7^ Disulfide bonds can undergo bond exchange reactions under external stimuli, such as heat or mechanical stress, enabling the reformation of damaged networks. However, disulfide-based materials often require thermal activation to achieve good self-healing performance.^8^ Recent studies have demonstrated that introducing aromatic units into the molecular structure can enhance thermal stability and stiffness through π-π stacking interactions while simultaneously reducing the energy barrier for disulfide bond exchange.^9,10^ In our previous work, we synthesized three distinct aromatic disulfide cross-linkers with low dissociation energy and then compared the molecular design and concentration of these cross-linkers to assess their impact on self-healing performance.^11^ To further improve recyclability and material performance, a new molecular design of an aromatic disulfide cross-linker is vital to optimizing the balance between self-healing efficiency, mechanical strength, and thermal stability.

In this work, three distinct aromatic disulfide-based cross-linkers were synthesized: Bis­[4-(methacrylyl-2-methyl-isocyanato-isophorone)­phenyl] disulfide (MIS), Bis­[4-(acrylyl-2-methyl-isocyanato-isophorone)­phenyl] disulfide (AIS), and Bis­[4-(methacrylyl-2-methylamide-isocyanato-isophorone)­benzyl amide] disulfide (MUS) (Scheme 1). Each cross-linker offers unique structural advantages. MIS and MUS contain an α-methacrylate group, which increases steric hindrance in their backbone structures of polymeric networks. AIS, on the other hand, features an acrylate moiety that can enhance flexibility. The urea group in MUS and urethane group in MIS and AIS forming by reacting with isophorone diisocyanate (IPDI) promote hydrogen bonding and crystallinity. These cross-linkers were designed to investigate the effects of molecular structure on thermal stability, mechanical strength, self-healing efficiency, and recyclability of the elastomers. Butyl acrylate (BA) monomer, widely used as a raw material in fiber processing agents, adhesives, coatings, plastics, acrylic rubber, and emulsions, serves as the basic building block of elastomers.^12^ Polyethylene glycol dimethacrylate (PEGDMA), a commercially available methacrylic cross-linker, was used to prepare the control sample in the same sample preparation process. The synthesis and purity of the cross-linkers were confirmed via ^1^H NMR, and the elastomers were subjected to a comprehensive set of characterizations, including thermogravimetric analysis (TGA), attenuated total reflectance Fourier-transform infrared (ATR-FTIR), differential scanning calorimetry (DSC), wide-angle X-ray scattering (WAXS), X-ray photoelectron spectroscopy (XPS), and mechanical tests. This study aims to elucidate the relationships between the molecular structure of cross-linkers and the physicochemical properties of (meth)­acrylic elastomers, providing insights into the design of recyclable and self-healing materials with tailored thermal and mechanical performance.

## Results and Discussion

### Synthesis and Characterization of Cross-linkers

The synthesis of MIS, AIS, and MUS was carried out through two-step reactions (Scheme S1). The structures of the cross-linkers were confirmed using ^1^H NMR spectroscopy (Figure S1-3). The proton signals in the NMR spectra for MIS, AIS, and MUS corresponded well with the expected chemical structures, and high-purity products were obtained with yields of 93% for MIS, 91% for AIS, and 83% for MUS. The data validated the successful synthesis of the targeted cross-linkers, confirming the presence of the appropriate functional groups necessary for their intended dynamic covalent structures.

**Scheme 1:**
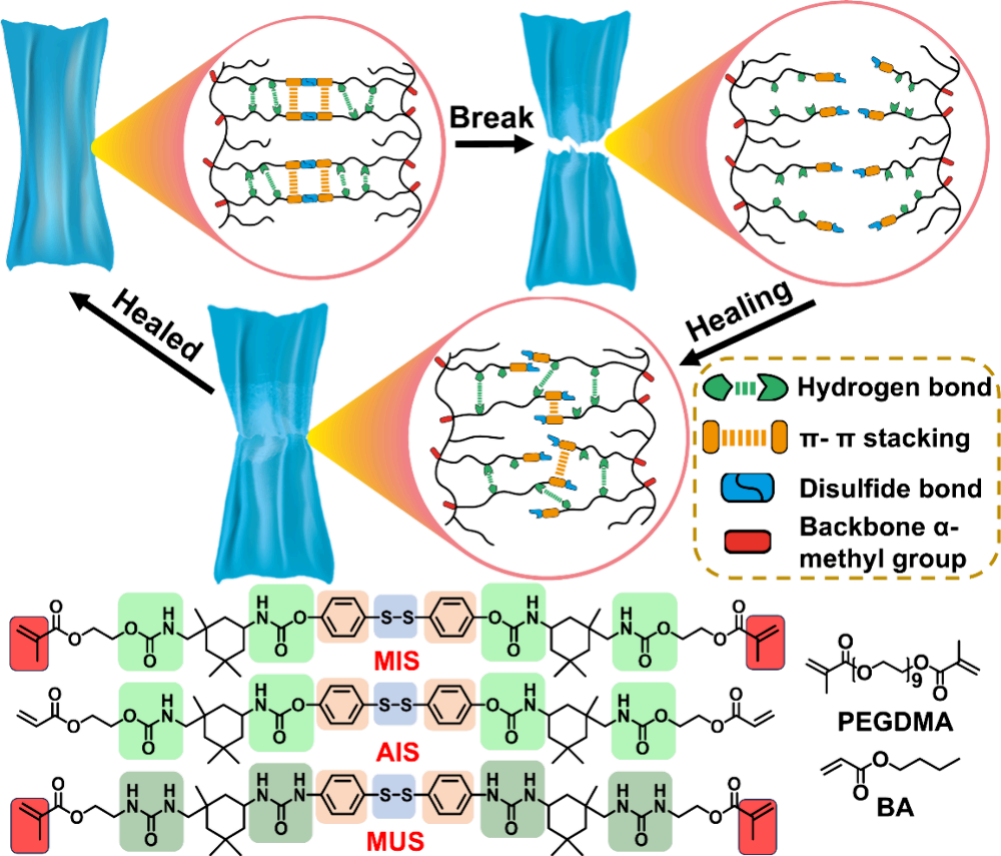
Chemical structures of MIS, AIS, MUS, PEGDMA and Cross-linkers, BA monomer, and the Self-Healing Elastomer Concept

TGA was used to assess the thermal stability of the cross-linkers (Figure S4a-c). The degradation profiles of the cross-linkers revealed three distinct degradation stages. The initial stage, corresponding to the cleavage of disulfide bonds, was observed at 274.8 °C for MIS, 266.5 °C for AIS, and 249.7 °C for MUS, which were caused by the relatively lower bond dissociation energy of the disulfide bond compared with the carbon–carbon bond.^13^ The second degradation stage occurred between 309.7 and 315.5 °C for all cross-linkers, corresponding to the breakdown of the urethane and urea moieties, leading to the generation of isocyanates and amines.^14^ In the final stage, which involved the decomposition of aromatic rings and the residual materials, degradation temperatures of 407.6 °C, 418.3 °C, and 355.2 °C were recorded for MIS, AIS, and MUS, respectively.^13^ The lower degradation temperature for MUS is attributed to the greater degree of crystallinity introduced by the urea moieties, which restricts chain mobility and increases susceptibility to thermal decomposition.^15^ In the thermal processing of elastomers or resins, the initial weight loss (≤5%) is typically attributed to the removal of moisture and volatile compounds from the sample. When the weight loss reaches 30%, it indicates that the temperature has caused nearly irreversible damage to the sample structure. Therefore, if the processing temperature of the sample is lower than the thermal degradation temperature at 30% weight loss, the material structure will not be significantly affected.^16^ As illustrated in Figure S 4d, all the cross-linkers have T_30%_ higher than 270 °C, which is suitable for most of the applications involving elastomer usage.

### Structural Characterization of Elastomer

Figure 1a represents the BA elastomers cross-linked by MIS, AIS, MUS, and PEGDMA cross-linkers. The elastomers of MIS-BA, AIS-BA, and PEGDMA-BA have high transparency, unlike the translucent MUS-BA. ATR-FTIR was employed to confirm the presence of key functional groups in the elastomers. In Figure 1b, the broad N–H stretching vibration related to hydrogen-bonded N–H at 3350 cm^–1^ and the free C = O stretching vibration at 1727 cm^–1^ were evident in all samples.^17^ There is a red shift of C = O stretch peak at 1640 cm^–1^ corresponding to hydrogen-bonded C = O. This peak of the MUS-BA sample exhibited a significantly higher intensity compared with MIS-BA and AIS-BA, indicating that more hydrogen bonding is present in MUS-BA, which further explains the translucency of the MUS-BA elastomer.^18^ The characteristic benzene ring vibrations were observed at 853 cm^–1^, confirming the presence of aromatic structures in the polymer backbone, which play a crucial role in the mechanical rigidity of the elastomers. Additionally, the C–N stretching vibrations at 1230 cm^–1^ and urethane-associated vibrations at 1530 cm^–1^ (amide II) and 1444 cm^–1^ (amide III) were consistent across all samples, further confirming the integrity of the cross-linker structure within the elastomer matrix.^17^

**Figure 1:**
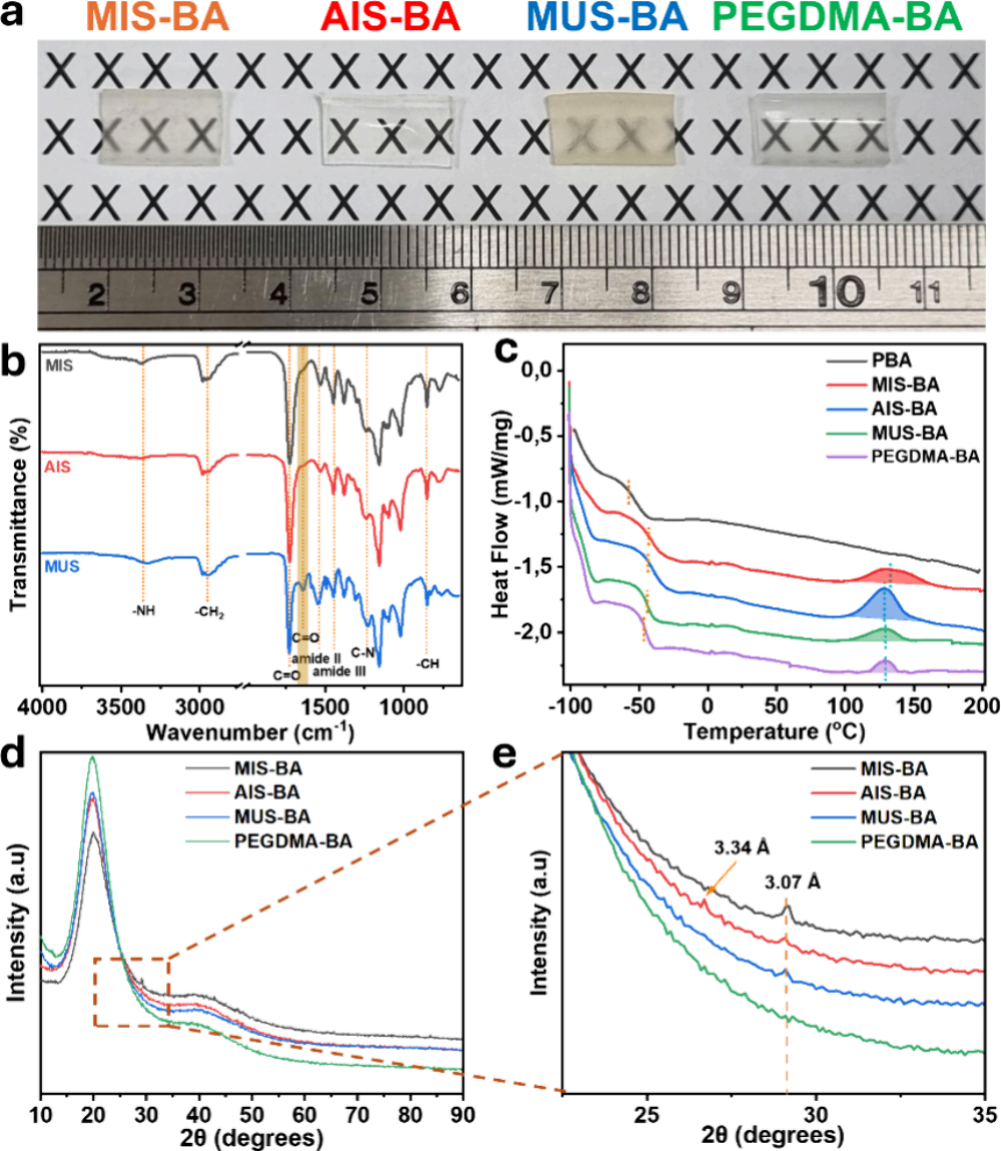
(a) Digital image of MIS-BA, AIS-BA, MUS-BA, and PEGDMA-BA elastomers; (b) ATR-FTIR spectra of MIS-BA, AIS-BA, and MUS-BA; (c) DSC thermograms of PBA, MIS-BA-AIS-BA, MUS-BA, and PEGDMA-BA; (d, e) WAXS patterns of MIS-BA, AIS-BA, MUS-BA, and PEGDMA-BA.

DSC analysis was employed to further confirm the critical role of the cross-linker in the elastomer properties. As shown in Figure S5, the linear poly­(butyl acrylate) (PBA) sample without cross-linker exists in a viscous conformation, in contrast to the other elastomer samples exhibiting a three-dimensional structure at room temperature. This indicates that the weak Val der Waals forces between butyl groups in PBA alone are insufficient to establish a stable three-dimensional structure. Figure 1c and Table S1 illustrate the DSC analysis. No endothermic or exothermic peaks can be observed in PBA. The PBA sample only has the *T*_g_ peak at −55 °C, which confirms the amorphous structure of PBA.^19^ The other elastomers have a *T*_g_ ranging from −46 to −44 °C, which is still significantly lower than room temperature. As a result, the cross-linked polymer chains in these elastomers exhibit high mobility, facilitating disulfide exchange at room temperature. In case of PEGDMA-BA, MIS-BA, AIS-BA, and MUS-BA, the exothermic peaks at 128 °C are attributed to the postcuring process of the elastomer during heating.^20^ When comparing the peak areas of the samples shown in Table S1, the order of increasing intensity is as follows: AIS-BA > MIS-BA > MUS-BA > PEGDMA-BA. This indicates that, besides the postcuring process, the heat release can be attributed to disulfide-thiol interactions and the crystallization process in AIS-BA, MIS-BA, and MUS-BA. MUS-BA with more hydrogen bonds in its structure, as indicated in FT-IR results, requires less energy for crystallization. In contrast, the polymer chains in AIS-BA are more flexible and demand more energy to develop crystalline structures within the material.^21^ The smaller exothermic peaks of MIS-BA and MUS-BA compared with AIS-BA also due to the steric hindrance imposed by α-methyl groups and excessive hydrogen bonding.^22,23^ This variation in crystallization contributed to differences in thermal stability and mechanical flexibility.

The wide-angle X-ray scattering (WAXS) patterns of the cross-linked elastomers revealed distinct features associated with π–π stacking interactions among the aromatic moieties in the network. In Figure 1d, all samples exhibit a broad diffraction peak centered around 2θ ≈ 19–21°, characteristic of an amorphous structure. As illustrated in Figure 1e, the samples containing aromatic cross-linkers exhibited a sharp diffraction peak centered at around 2θ ≈ 29°, corresponding to an interplanar distance of ∼ 3.07 Å, which is characteristic of face-to-face π–π stacking interactions.^24^ Notably, the AIS-BA sample displayed an additional peak at 2θ ≈ 26.6°, equivalent to a *d*-spacing of 3.34 Å, absent in the other cross-linked systems. This larger interplanar distance suggests a more offset or looser π–π stacking geometry, potentially arising from the higher conformational flexibility of the acrylate side groups in AIS compared to the bulkier methacrylate groups in MIS and MUS.^25^ The difference in side-group bulk and flexibility likely influences the supramolecular packing of the aromatic units, leading to variations in local order and stacking distances. These subtle differences in stacking geometry could also play a role in modulating the mechanical and self-healing properties of the resulting materials, as π–π stacking contributes to reversible physical cross-linking and network dynamics.^26,27^

XPS was utilized to analyze the surface composition and chemical states of sulfur and nitrogen atoms in the elastomer samples (Figure 2). Table S2 presents the ratios of surface disulfide (S–S) and thiol (S–H) groups before and after surface scratching for MIS-BA, AIS-BA, and MUS-BA. Before scratching, all samples exhibited 100% disulfide content on the surface, indicating the stability of the disulfide bonds in the elastomer network (Figure 2 **a-c**). However, significant differences are observed in the thiol-to-disulfide ratios among the samples after scratching. In the cross-section areas, MIS-BA shows 55.3% of thiol and 44.7% of disulfide contents; AIS-BA exhibits 62.9% of thiol and 36.1% of disulfide. Meanwhile, MUS-BA has a balanced ratio of thiol and disulfide bonds.

**Figure 2:**
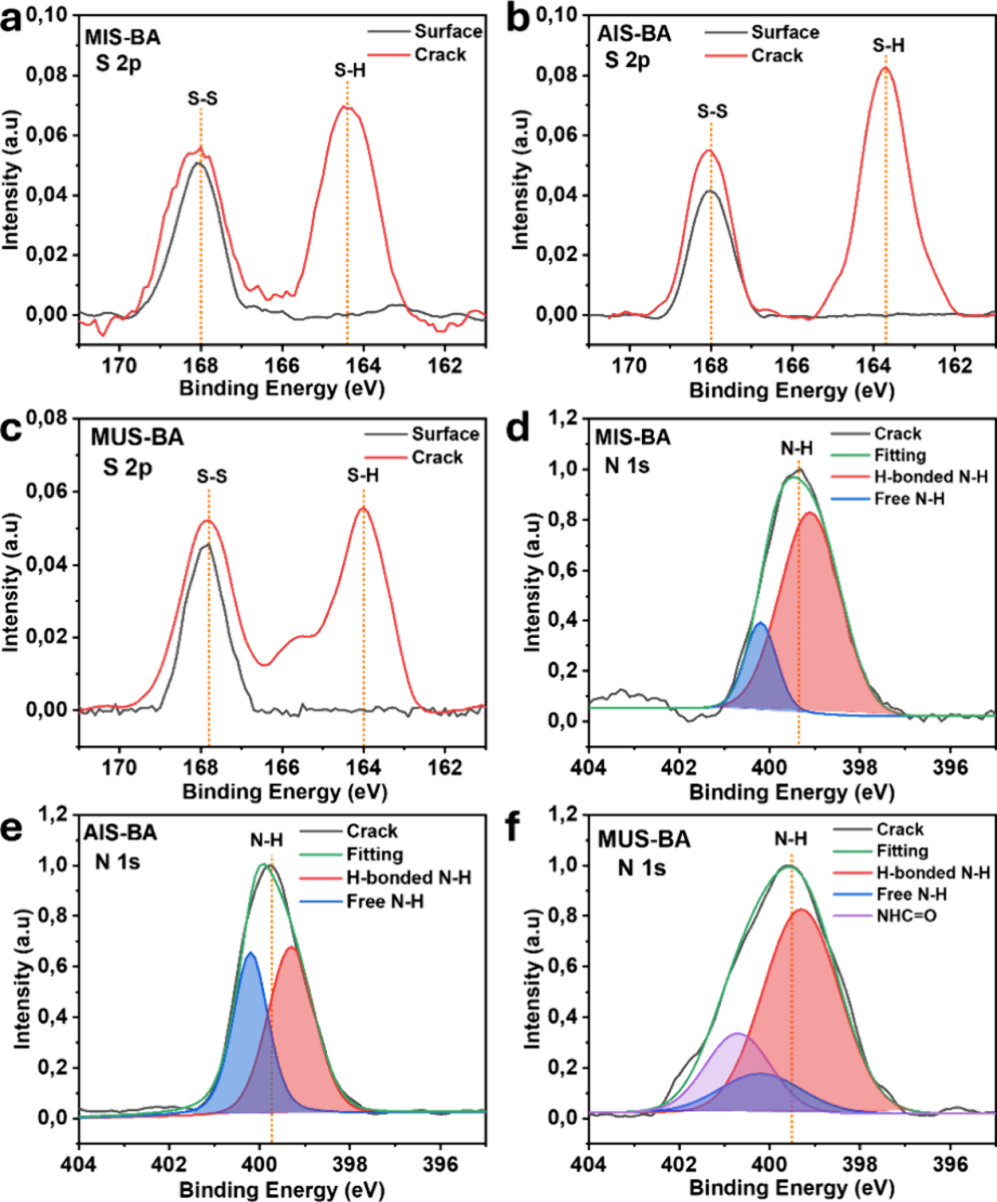
XPS sulfur spectra of (a) MIS-BA, (b) AIS-BA, and (c) MUS-BA; XPS nitrogen spectra and fitting curves of (d) MIS-BA, (e) AIS-BA, and (f) MUS-BA.

These variations in surface composition can be attributed to the distinct mobility and exchange capabilities of the disulfide bonds. With the flexible acrylate backbone, AIS-BA demonstrates the highest thiol content in the damaged area, indicating high chain mobility and efficient disulfide cleavage under mechanical forces. In contrast, the high steric hindrance caused by the methyl groups in the backbones of MIS-BA and MUS-BA reduces disulfide cleavage, resulting in a lower thiol concentration.^28^ In addition, the increased rigidity of the molecular chains caused by the presence of a higher density of hydrogen bonding interactions in the MUS structure reduced the cleavage of disulfide bonds at the damaged region.

The nitrogen spectra further reveal the types and relative proportions of nitrogen-containing groups in the elastomers, as detailed in Figure 2 **d-f** and Table S3.^29^ MIS-BA primarily contains hydrogen-bonded N–H groups (0.244 atom %) and a smaller amount of free N–H groups (0.055 atom %). In contrast, AIS-BA has a lower proportion of hydrogen-bonded N–H (0.191 atom %) but a higher ratio of free N–H (0.148 atom %), supporting the enhanced chain mobility observed in its disulfide-thiol exchange. MUS-BA, due to the formation of urea structures, shows a distinct NHC = O peak (0.083 atom %) in addition to hydrogen-bonded (0.255 atom %) and free N–H groups (0.051 atom %).

These findings demonstrate that the dynamic exchange reactions between thiol and disulfide bonds are significantly influenced by various functional groups and structural characteristics. The α-methyl groups in MIS-BA hinder exchange efficiency, whereas the balanced hydrogen bonding and acrylate groups in AIS-BA facilitate improved disulfide-thiol metathesis, beneficial to surface self-healing performance. In contrast, the distinctive urea structure in MUS-BA introduces additional steric hindrance, thereby compromising the self-healing efficiency of MUS-BA.

### Self-Healing Efficiency

Self-healing efficiency was evaluated by observing and quantifying the recovery of damaged samples at room temperature within 24 h, then comparing them with the original samples. The first one is surface healing efficiency, illustrated in Figure 3. PEGDMA-BA presents no sign of healing after 24 h because of the absence of dynamic bonds. AIS demonstrated the highest self-healing efficiency, reflecting an optimal balance between chain mobility and hydrogen bonding, facilitating efficient disulfide bond exchange. Although MIS-BA and AIS-BA exhibited similar glass transition temperatures, the AIS-BA elastomer demonstrated significantly higher self-healing performance. This can be attributed to the chemical structure of the acrylate-based cross-linker (AIS), which lacks the steric hindrance of the α-methyl group found in methacrylates (MIS). As a result, AIS-BA forms a more flexible and dynamically responsive polymer network, facilitating chain mobility and the reformation of noncovalent interactions (e.g., π–π stacking and hydrogen bonding) after damage. The enhanced molecular mobility, even in the absence of a Tg shift, promotes more efficient surface and bulk self-healing. MIS exhibited a moderate self-healing efficiency, limited by the steric hindrance of the backbone α-methyl groups that restricted chain mobility and hindered disulfide reformation.^30^ MUS showed the lowest self-healing efficiency between the disulfide cross-linkers, likely due to the high degree of hydrogen bonding that restricted the dynamic exchange of disulfide bonds, thereby preventing effective self-repairing.^31^

**Figure 3:**
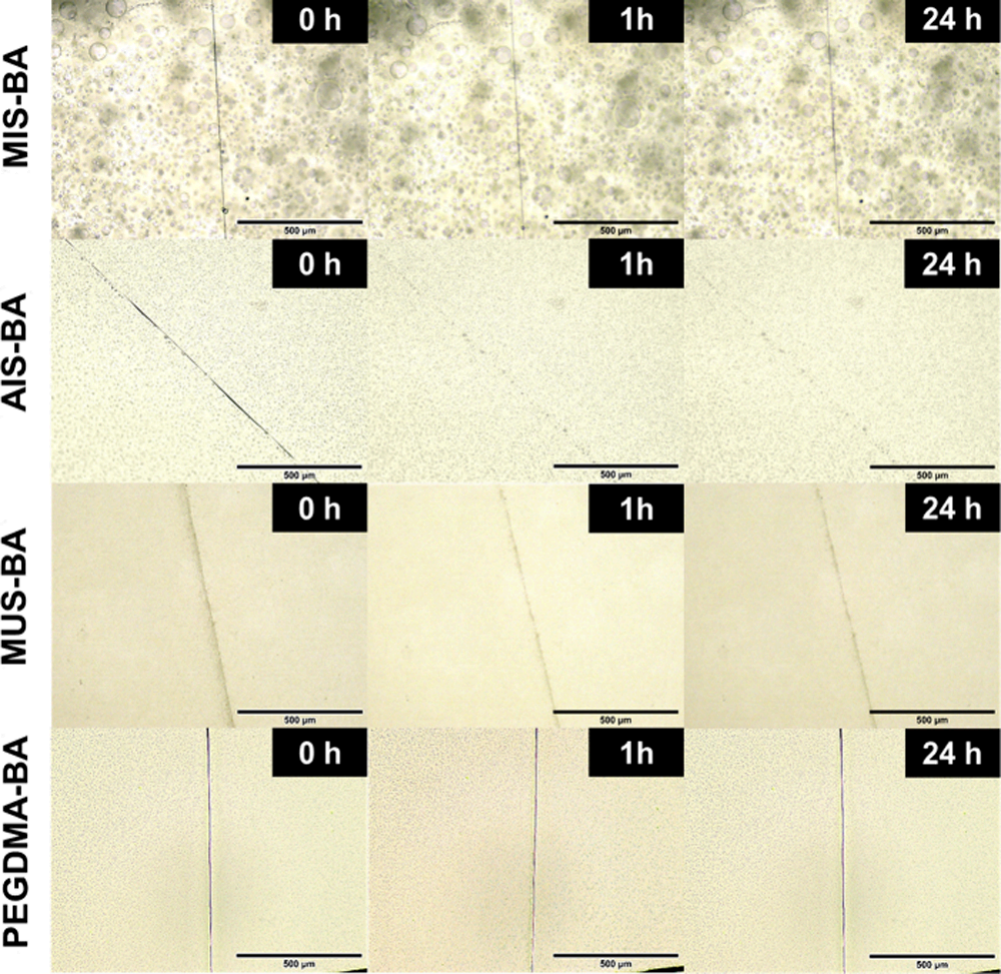
Optical microscope images of scratches on elastomers.

Figure 4a illustrates the self-healing ability of AIS-BA. The AIS-BA piece was cut, one slice was dyed, and the two slices were allowed to contact each other at room temperature. After 24 h, the healed elastomer can support a 30-g bottle without tearing. Additionally, the dye did not migrate to the other slice, indicating that the healing process occurs solely through the reformation of bonds, such as disulfide bonds, hydrogen bonds, and π–π stacking, rather than through the migration of polymer chains. For the recovery of mechanical properties, the UTS of MIS-BA, AIS-BA, and MUS-BA before and after self-healing were compared to those of PEGDMA-BA to evaluate the influence of cross-linker molecular structure on the mechanical properties of the resultant elastomers. The cross-linking density was calculated from the elastic modulus. MIS-BA and AIS-BA have almost the same cross-linking density (14.62 and 12.27 mol/m^3^), while MUS-BA has twice the amount of cross-linking density compared with MIS-BA and AIS-BA (24.62 mol/m^3^), despite the same cross-linker content being used in the elastomer preparation process. This is because hydrogen bonds act as temporary cross-linking agents, which increase the elastic modulus and render the calculated cross-linking density based on the elastic modulus inaccurate. Moreover, the elastomer preparation process, which involves thermal polymerization at 50 °C for a time longer than 12 h, along with heating at 60 °C for 24 h, can ensure high monomer/cross-linker conversion. As indicated in Figure 4 **b-e**, due to the presence of benzene rings, urethane moiety in MIS, AIS, and urea moiety in MUS cross-linkers, which impart rigid aromatic hydrocarbon structures, conjugation effects, and hydrogen bonding, these samples exhibit significantly higher ultimate tensile strength (UTS) (Figure 4f), toughness (Table S4), and elongation at break compared to PEGDMA-BA.^28,32^ This indicates the increased stiffness and strength provided by the π-π stacking and hydrogen bonds. When comparing the tensile test curves of MIS-BA and AIS-BA, AIS-BA showed a slightly higher elongation at break, which is attributed to the greater chain flexibility in AIS-BA. In contrast, the α-methacrylate groups in MIS-BA impose steric hindrance in hydrophobic regions, restricting polymer chain mobility and reducing elongation.^33^ The steric hindrance effect of the backbone α-methyl groups also interferes with the formation of intermolecular hydrogen bonds, further impacting the rigidity and flexibility of the chain segments.^30^ MUS-BA, however, demonstrated the highest UTS and toughness, highlighting the positive impact of increased intermolecular hydrogen bonding on mechanical performance. A higher number of hydrogen bonds in the MUS-BA network strengthens the intermolecular interactions, enhancing material toughness and strength. The improved mechanical properties can be linked to the formation of dynamic interfacial hydrogen bonding structures, which create a physically cross-linked network that provides higher strength. Additionally, the networking structure restricts the movement of urethane or urea segments within the cross-linkers, leading to a considerable enhancement in UTS and the overall toughness of MUS-BA. These findings suggest that adjusting the degree of hydrogen bonding and chain flexibility is crucial for optimizing the tensile performance of elastomers.

**Figure 4:**
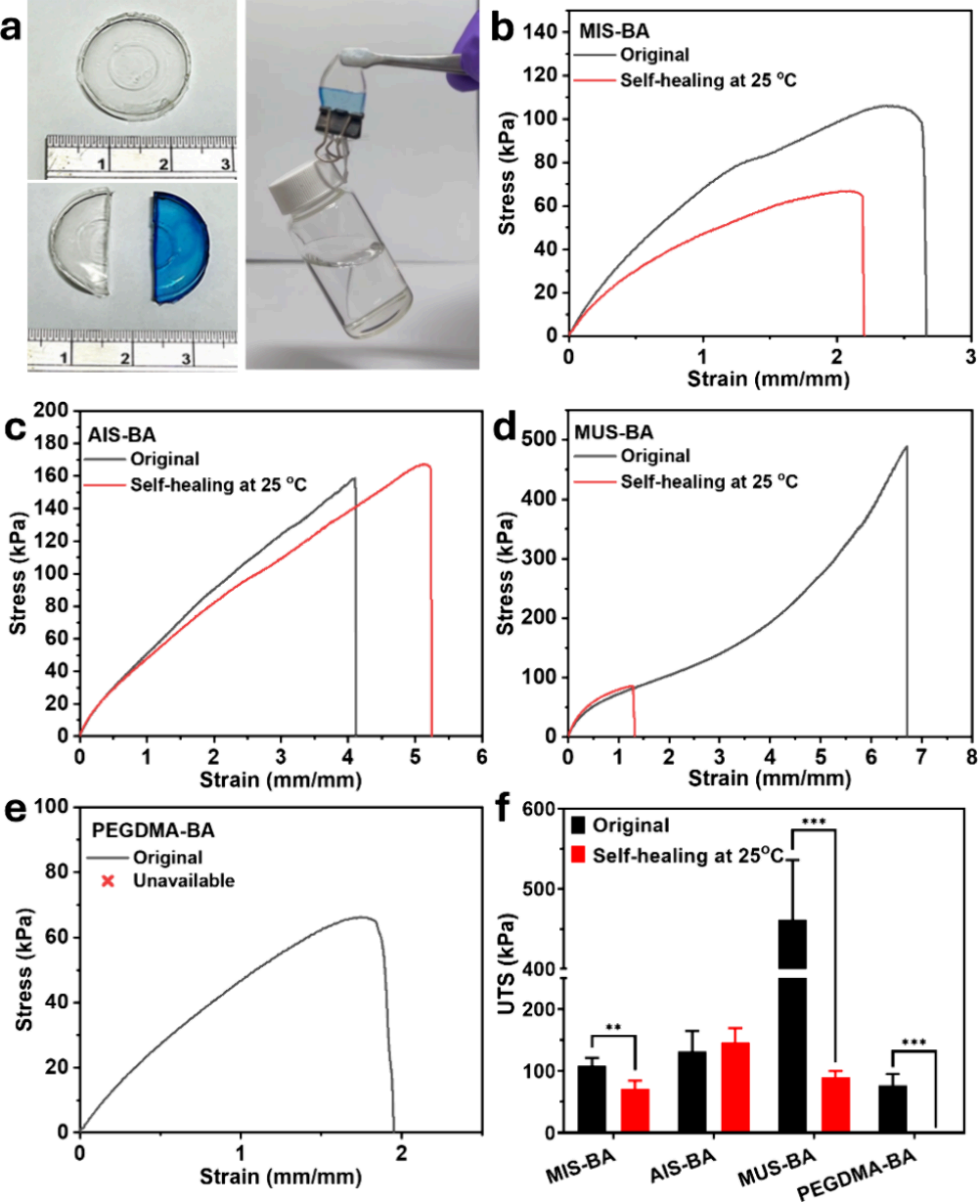
(a) Digital image of cut-healed AIS-BA elastomer, (b) tensile test of original and healed (b) MIS-BA, (c) AIS-BA, (d) MUS-BA, and (e) PEGDMA-BA; (f) UTS of original and healed elastomers. The values represent mean ± SD, *n* = 5.

The self-healing performance of synthesized elastomers was investigated to determine the impact of varying molecular structures on the ability to recover mechanical strength after damage. The results show distinct differences in self-healing efficiency among the elastomers (Figure 4 **b-f** and Tables S4 and S5). AIS-BA exhibits the highest mechanical property recovery, achieving 111% recovery, while MIS-BA has a moderate self-healing efficiency of 65.64%. In contrast, MUS-BA and PEGDMA-BA show significantly low self-healing efficiencies of 19.56% and 0%, respectively.

MIS-BA, AIS-BA, and MUS-BA can self-heal mainly due to the presence of dynamic disulfide

bonds, which undergo reversible exchange reactions and disulfide metathesis, facilitating efficient damage repair and reformation of the polymer network.^34^ Additionally, the π-π stacking interactions between the benzene ring structures further contribute to repairing damaged sites, enabling the elastomer to regain its mechanical strength.^35^ The hydrogen bond also acts as a healing factor in the elastomer. Notably, the superior self-healing performance of AIS-BA can be attributed to the acrylate group, which enhances the mobility of the polymer chain and the balanced strength of hydrogen bonds. These cooperative mechanisms result in the exceptional self-healing efficiency of AIS-BA. MIS-BA, on the other hand, shows lower self-healing efficiency compared to AIS-BA. This reduction is primarily due to the steric hindrance imposed by the backbone α-methyl groups, which not only reduce the formation of intermolecular hydrogen bonds but also limit the flexibility of the polymer backbones, thereby lowering the efficiency of energy dissipation and healing.^33^ MUS-BA, despite having multiple intermolecular hydrogen bonds, shows poor self-healing efficiency. The excessive number of hydrogen bonds can restrict molecular mobility, forming more crystalline regions within the structure, which negatively impacts healing capacity.^31^ Moreover, stronger hydrogen bonding demands higher energy for dissociation/reformation, diminishing the ability to recover from material damage. Overall, the results reveal that the type and interaction of functional groups significantly influence the self-healing behavior of elastomers, with disulfide bonds and π-π stacking interactions enhancing self-healing. At the same time, steric hindrance and excessive hydrogen bonds reduce recovery efficiency.

### Degradation and Recycling Process

The degradation and recyclability of the synthesized elastomers were examined to evaluate their potential for sustainable applications (Figure 5 **and** Figure S6). The chopped pieces of MIS-BA, AIS-BA, and MUS-BA can be wholly degraded in 1 M dithiothreitol (DTT) in THF solution at 100 °C after 7 days, while PEGDMA-BA elastomer remains intact. The degradation mechanism is primarily governed by the dynamic nature of the disulfide bonds, which can undergo reversible bond exchange reactions in response to both reducing agents and thermal stimuli.^36^ Reduction experiments using DTT revealed that the generated disulfide-bonded 6-membered ring is highly stable, indicating the reaction does not produce mixed disulfides and maintains the integrity of the resultant fragments.^37^ The stability suggests that the DTT reduction effectively cleaves the disulfide linkages without interfering with other functional groups in the polymer, ensuring a clean degradation process. Furthermore, the disulfide bonds exhibit a dual sensitivity, reacting not only with reductants but also undergoing degradation at elevated temperatures. ^1^H NMR of the degradation solution confirms the presence of DTT, threitol disulfide (the oxidized form of DTT), and the polymer chain that degraded from MIS-BA, AIS-BA, and MUS-BA (Figure S7-9). There are no peaks of double bonds from monomers or cross-linkers at 5.5–6.5 ppm, indicating that no unreacted monomers or cross-linkers remain in the elastomers. The results clarify that DTT reduces the disulfide bonds in these elastomers, disrupting their polymer networks and rendering them soluble in a suitable solvent. Thermal degradation is significantly influenced by the interaction of the disulfide bonds in a suitable solvent for the degraded products. At high temperatures, the solvent facilitates the diffusion of solutes within the polymer matrix, thereby promoting the exchange reactions between thiol and disulfide bonds, accelerating the breakdown of the cross-linked network.^36^ The thermally induced bond exchange mechanism enhances the degradation rate, making the polymer more amenable to recycling under appropriate conditions. After degradation, water precipitation removes any residual DTT, ensuring the purity of the degraded fragments for subsequent reuse or recycling (Figure 5).^38^ The polymer can be collected and dissolved in THF. After THF evaporates, the elastomer forms into one piece again by reforming disulfide bonds. The study highlights that the elastomers can be efficiently degraded and recycled through controlled thermal and chemical treatments, thus supporting their potential for sustainable applications.

**Figure 5:**
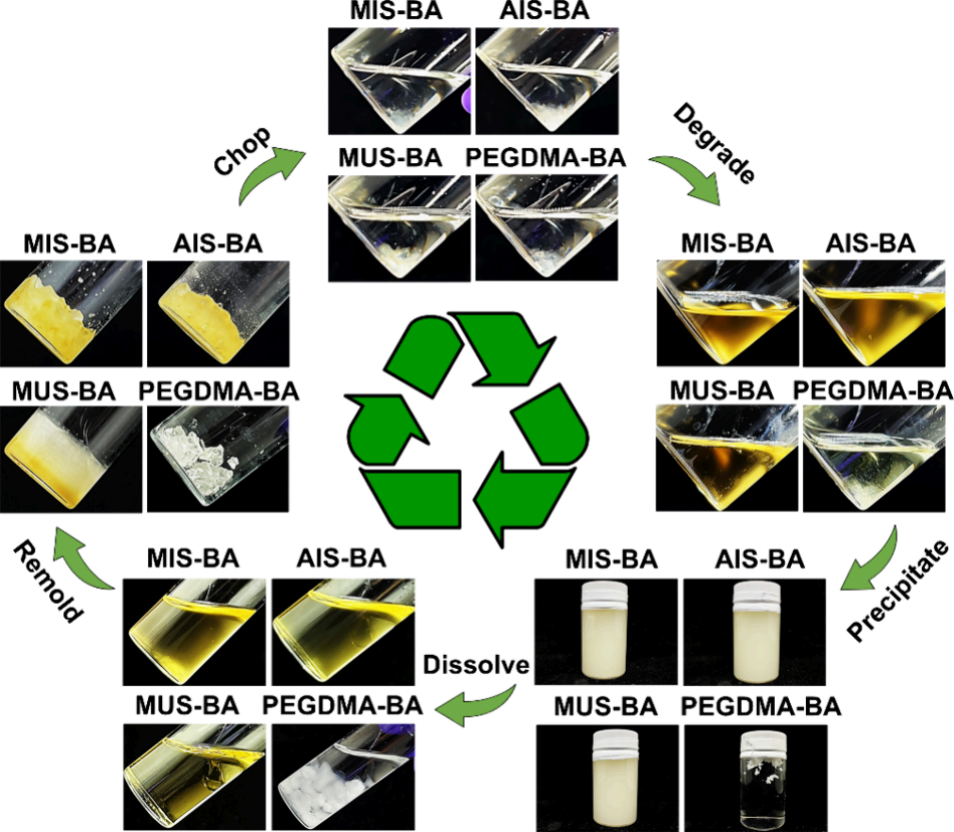
Illustration of the elastomer recycling process.

## Conclusions

This study provides a detailed investigation into the influence of the molecular structure of aromatic disulfide-based cross-linkers on the thermal, mechanical, and self-healing properties of acrylic elastomers. The successful synthesis of MIS, AIS, and MUS cross-linkers was confirmed by ^1^H NMR, and their incorporation into elastomers resulted in materials with diverse thermal and mechanical properties. AIS, with internal dynamic aromatic disulfide, intermolecular secondary bonds, and balanced chain flexibility, exhibited the highest self-healing efficiency and moderate mechanical strength, making it a promising candidate for applications requiring both durability and self-healing properties. MIS provided high tensile strength but was limited in self-healing efficiency due to steric hindrance. Although offering superior mechanical strength, MUS demonstrated poor self-healing performance due to the high degree of crystallinity within the cross-linkers, which restricted disulfide bond exchange. The findings highlight the critical role of molecular structure in optimizing the performance of dynamic acrylic cross-linkers. It should be noted that the developed dynamic cross-linkers can be applied for various commercially available (meth)­acrylic monomers to endow improved mechanical, self-healing and recyclable properties. Future studies could explore modifications to the cross-linker design to enhance mechanical and self-healing properties, paving the way for advanced applications in innovative materials, coatings, and adhesives.

## Experimental Section

### Materials

4,4’-dihydroxydiphenyl disulfide (SS), 2-isocyanoethyl acrylate (IEA), 2-isocyanoethyl methacrylate (IEMA), 4-Aminophenyl disulfide (4-AFD), and Dibutyltin dilaurate (DBTDL) were purchased from Tokyo Chemical Industry (TCI). Isophorone diisocyanate (IPDI), Hydroxyethyl methacrylate (HEMA), Hydroxyethyl acrylate (HEA), and 2-aminoethyl methacrylate hydrochloride (AMA) were purchased from Acros Organics. Two 2-Azobis­(isobutyronitrile) (AIBN), and Dimethylacetamide (DMAc) were purchased from CORE Chemical Inc. Butyl acrylate (BA) were purchased from Alfa Aesar. Polyethylene glycol dimethacrylate (PEGDMA, 550 Da), and Dimethylformamide (DMF) were purchased from Sigma-Aldrich. Tetrahydrofuran (THF, anhydrous), and Acetone were purchased from TEDIA. Dimethyl sulfoxide (DMSO) were purchased from ECHO CHEMICAL CO., Ltd. Dithiothreitol (DTT) were purchased from Bio-Tech. Deionized water was obtained using a Milli-Q system (Millipore, Bedford, MA, USA). All the other chemicals were of reagent grade and used as received without further purification.

### Synthesis of Methacrylate/Acrylate Isophorone-Based Disulfide (MIS/AIS)

4,4’-Dihydroxydiphenyl disulfide (SS) (2 mmol) was dissolved in THF (5 mL) and heated to 50 °C under nitrogen. A solution of isophorone diisocyanate (IPDI) (6 mmol) and dibutyltin dilaurate (DBTDL) (2000 ppm) in THF (5 mL) was added dropwise, and the reaction proceeded for 5 h. Next, hydroxyethyl methacrylate (HEMA) or hydroxyethyl acrylate (HEA) (8 mmol) in THF (5 mL) was introduced dropwise, followed by another 5-h reaction. After vacuum concentration, the crude product was precipitated in deionized water, redissolved in acetone, and vacuum-dried, yielding a white (MIS) or light yellow (AIS) powder.

### Synthesis of Methacrylate Urea-Based Disulfide (MUS)

4-Aminophenyl disulfide (4-AFD) (2 mmol) was dissolved in DMF (5 mL) and cooled to 0 °C in an ice bath under nitrogen. A solution of isophorone diisocyanate (IPDI) (6 mmol) and dibutyltin dilaurate (DBTDL) (2000 ppm) in DMF (5 mL) was added dropwise. After 10 min of stirring, the mixture warmed to room temperature, then heated to 60 °C for 5 h under nitrogen. The reaction was cooled to 0 °C, followed by the dropwise addition of 2-aminoethyl methacrylate hydrochloride (AMA) (8 mmol) in DMF (5 mL), and the reaction proceeded for another 2 h. The final product was precipitated in deionized water and then vacuum-dried, yielding a light brown powder.

### BA Elastomer Preparation

The thermal initiator 2,2’-azobis­(isobutyronitrile) (AIBN) (1 mol % relative to the monomer) was dissolved in THF for MIS-BA and AIS-BA, while DMSO was used as the solvent for the MUS-BA preparation. The solution was purged with nitrogen for 30 min to eliminate oxygen. The ratio of cross-linkers is 1%, and the total concentration of monomers and cross-linkers was maintained at 3 M (6 M for PBA samples). The mixture was prepared under an argon atmosphere and cast into polypropylene or polytetrafluoroethylene molds. Polymerization was carried out by heating the reaction mixture at 60 °C overnight. Following polymerization, the elastic samples were further dried at 60 °C for 24 h to remove residual solvents.

### Thermal Characterization

Thermogravimetric analysis (TGA, TA Q500, TA Instruments) was performed to assess the thermal stability of the cross-linkers under a nitrogen atmosphere, using a heating rate of 10 °C/min from 20 to 500 °C. Differential scanning calorimetry (DSC) was conducted using an LT-DSC (Netzsch 204 F1, NETZSCH Instruments) under nitrogen flow. Samples (8–10 mg) in standard DSC pans were cooled to – 100 °C and subsequently heated to 200 °C at 10 °C/min.

### Self-Healing Behavior Analysis

Self-healing behavior was examined using an optical microscope (Nikon Eclipse Ts2-FL, Tokyo, Japan) at 10× magnification. Scratched cross-linked elastomer samples were analyzed, and surface healing efficiency was quantified based on the change in scratch area using Equation 1):

$$\mathrm{Surface}\;\mathrm{healing}\;\mathrm{efficiency}\left(\%\right)=\frac{\left(\mathrm{Initial}\;\mathrm{area}-\mathrm{Final}\;\mathrm{area}\right)}{\mathrm{Initial}\;\mathrm{area}}x100$$ 1

Additionally, mechanical property recovery was evaluated via tensile testing. Dumbbell-shaped specimens (15 mm × 5 mm × 2 mm) were subjected to tensile testing using a universal tensile testing machine (QC-508M1F, ComeTech, Taiwan) at ambient temperature and a stretching speed of 100 mm/min. Each tensile test was conducted on an average of five specimens. The stress–strain data from both pristine and healed specimens were used to calculate mechanical property recovery via Equation 2):

$$\mathrm{Mechanical}\;\mathrm{property}\;\mathrm{recovery}\left(\%\right)=\frac{\mathrm{Recovery}\;{\mathrm{UTS}}_{\left(n\right)}}{\mathrm{Original}\;\mathrm{UTS}}\times 100$$ 2

Where the recovery UTS_(n)_ and the original UTS are the average UTS of the healed specimens and the original specimens, respectively.

### Recycling Test

The elastomer samples were cut into specimens measuring 3 cm in length, 1 cm in width, and 0.1 cm in thickness. These specimens were then ground into powder and immersed in THF to achieve swelling equilibrium. Subsequently, DTT was introduced into the solvent containing the swollen elastomer to reach 1 M in concentration, and the dissolution behavior was monitored at 100 °C. A small amount of solution was taken from each bottle for ^1^H NMR test. Following reduction, the solvent phase was precipitated in water, and the resulting precipitate was redissolved in THF. Finally, THF was removed by oven drying, and the reformation process of the elastomer was analyzed.

### Other Characterizations

^1^H Nuclear Magnetic Resonance (^1^H NMR) spectra were recorded using a Bruker AVANCE III HD 600 MHz NMR, with chemical shifts referenced to trimethylsilane (TMS) at 0 ppm. ATR-FTIR spectra were measured using Shimadzu IR Spirit. X-ray photoelectron spectroscopy (XPS) measurements were conducted on a Theta Probe AR-XPS system (Thermo Fisher Scientific, UK) with a monochromatic Al Kα X-ray source (1486.6 eV, 15 kV) and a 50 μm beam spot size. Wide-angle X-ray scattering (WAXS) was measured using a Bruker X-ray diffractometer equipped with a diffracted beam monochromator and Cu Kα radiation at 40 kV and 100 mA. The scan range is from 10 to 90° 2θ, with a step width of 0.1° 2θ, and a scan rate of 0.5° 2θ per minute. The divergent slit is set at 1/6° and the receiving slit is 0.3 mm. Data were reported as mean ± SD (*n* = 5) and analyzed using Student’s *t* test and one-way ANOVA, with statistical significance set at *p* ≤ 0.05. All statistical analyses were performed using GraphPad Prism 9.0 (GraphPad Software, USA).

## Supplementary Material



## Notes

Synthesis routes scheme, ^1^H NMR spectra for all compounds, and additional experimental details (PDF)
