# Supplementary material for: Molecular Design in Dynamic (Meth)acrylic Cross-linkers for Tough, Self-healing, and Recyclable Elastomer
Source: Macromolecules. 2025 Jul 16;58(15):7967–74. doi: 10.1021/acs.macromol.5c00649 (PMC12356062; doi:10.1021/acs.macromol.5c00649)
Supplement: Supplementary file 1 [file ma5c00649_si_001.pdf]

Supporting Information

Molecular design in dynamic (meth)acrylic crosslinkers for tough, self-healing and recyclable elastomer

Hoang Nam Nguyen[#], Li-Hung Lu[#], Kai Ou, Doan Van Hong Thien, and Chun-Jen Huang*

Hoang Nam Nguyen - Faculty of Automation Engineering, Can Tho University, 3/2 Street, Ninh Kieu District, Can Tho City 900000, Vietnam.

Li-Hung Lu - Department of Chemical & Materials Engineering, National Central University, Jhong-Li, Taoyuan 32023, Taiwan.

Kai Ou - Department of Chemical & Materials Engineering, National Central University, Jhong-Li, Taoyuan 32023, Taiwan.

Doan Van Hong Thien - Faculty of Chemical Engineering, Can Tho University, 3/2 Street, Ninh Kieu District, Can Tho City 900000, Vietnam.

Chun-Jen Huang - Department of Chemical & Materials Engineering, National Central University, Jhong-Li, Taoyuan 320, Taiwan; R&D Center for Membrane Technology, Chung Yuan Christian University, 200 Chung Pei Rd., Chung-Li City 32023, Taiwan; School of Materials Science and Engineering, The University of New South Wales, Sydney, New SouthWales 2052, Australia.

* Corresponding author: E-mail: cjhuang@ncu.edu.tw

# Equal contribution

Table S1. Physical properties of elastomeric polymers.

Sample	Glass transition Temperature (Tg, °C)	Crystallization temperature (Tc, °C)	Peak Area [J/g]
MIS-BA	-43	128.8	22.71
AIS-BA	-43.7	128.7	36.13
MUS-BA	-44.4	128.9	12.46
PEGDMA-BA	-46.7	128.7	6.59

Table S2. Proportions of surface disulfide and thiol functional groups in the elastomer.

Sample	Surface Atom Ratio (%)		Crack Atom Ratio (%)	
	Disulfide (S-S)	Thiol (S-H)	Disulfide (S-S)	Thiol (S-H)
**MIS-BA**	100	0	44.7	55.3
**AIS-BA**	100	0	36.1	62.9
**MUS-BA**	100	0	49.2	50.8

Table S3. The areas, widths at half maximum (FWHM), and atomic ratios of the XPS nitrogen spectral peaks in the elastomers.

Sample	Assignment	FWHM	Area	Atom ratio(%)
**MIS-BA**	Hydrogen bond	1.5625	1.3228	0.244
	Free N-H	1.3216	0.2998	0.055
**AIS-BA**	Hydrogen bond	1.2217	0.8902	0.191
	Free N-H	0.9257	0.6902	0.148
**MUS-BA**	Hydrogen bond	2.0176	1.6606	0.255
	Free N-H	2.3431	0.3313	0.051
	NHC=O	1.7651	0.5386	0.083

Table S4. Toughness of elastomers

Sample	Toughness (kJ/m^3)	
	Originial	Self-healed at 25°C
MIS-BA	189.2	99.3
AIS-BA	362.5	504.8
MUS-BA	1280.3	77
PEGDMA-BA	75.5	No value

Table S5. Self-healing efficiency of elastomers.

Sample	Self-healing capability (%)
MIS-BA	65.64
AIS-BA	111.0
MUS-BA	19.56

PEGDMA-BA	0

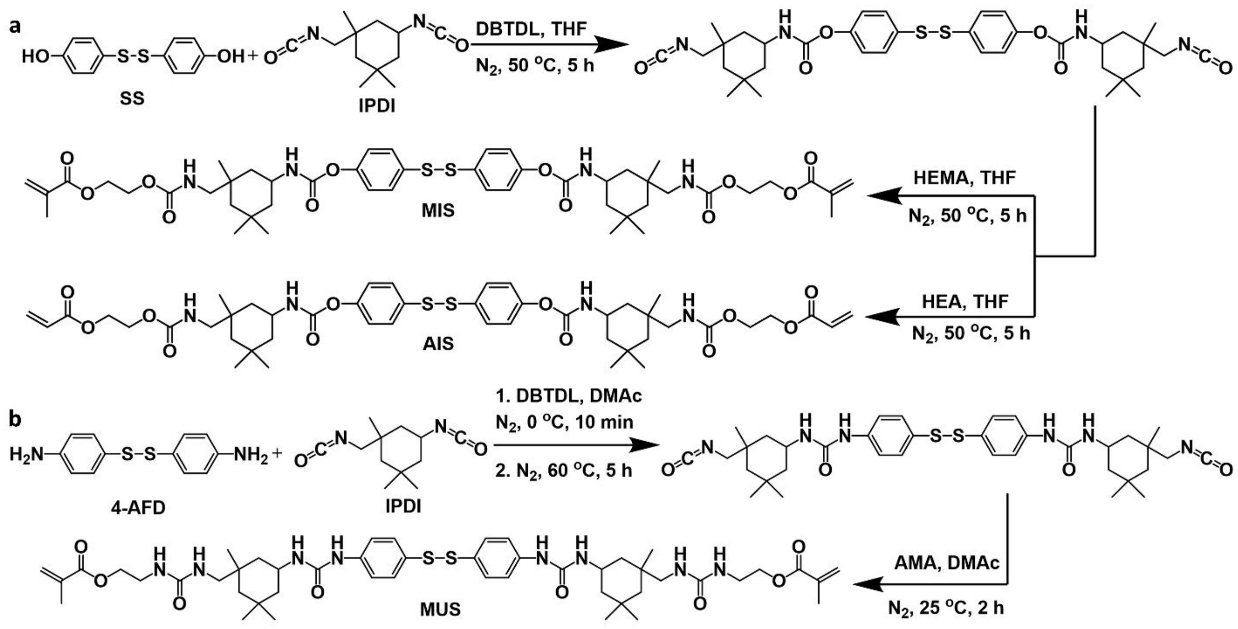

Scheme S1. Synthesis route of a) MIS and AIS, and b) MUS.

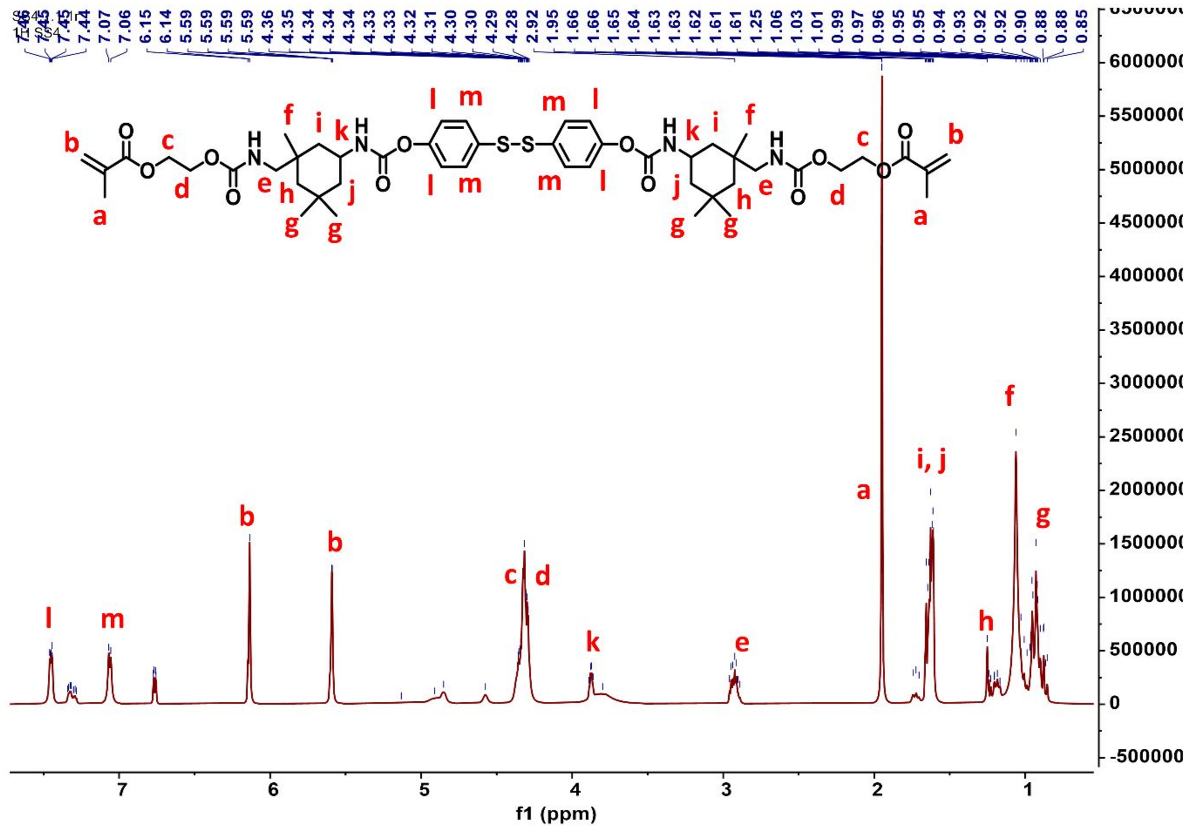

Figure S1. ^{1}H-NMR spectra of MIS crosslinker.

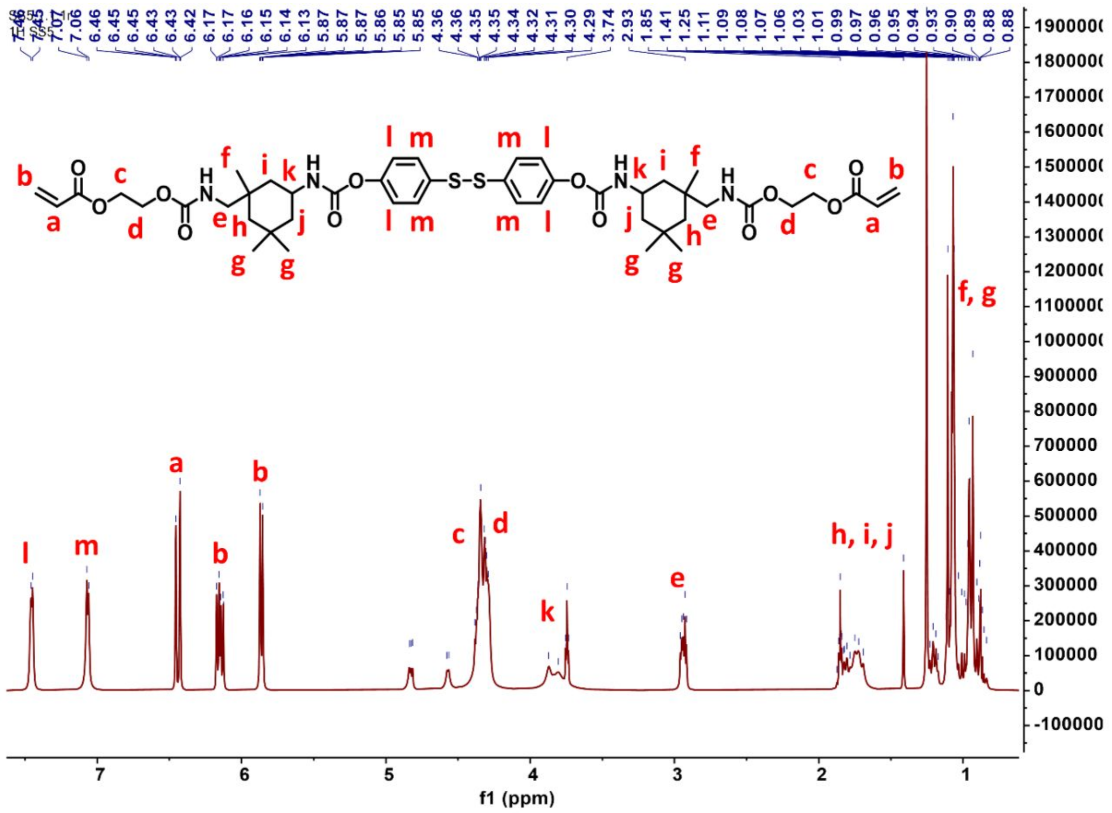

Figure S2. ^{1}H-NMR spectra of AIS crosslinker.

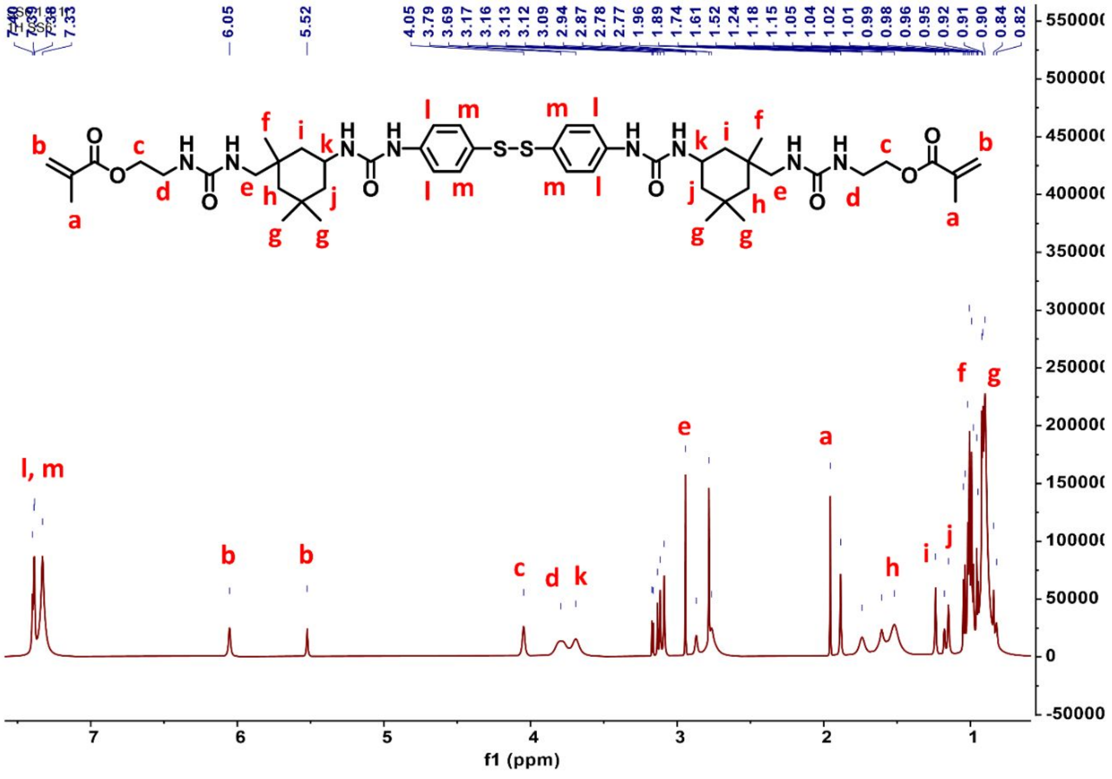

Figure S3. ^{1}H-NMR spectra of MIS crosslinker.

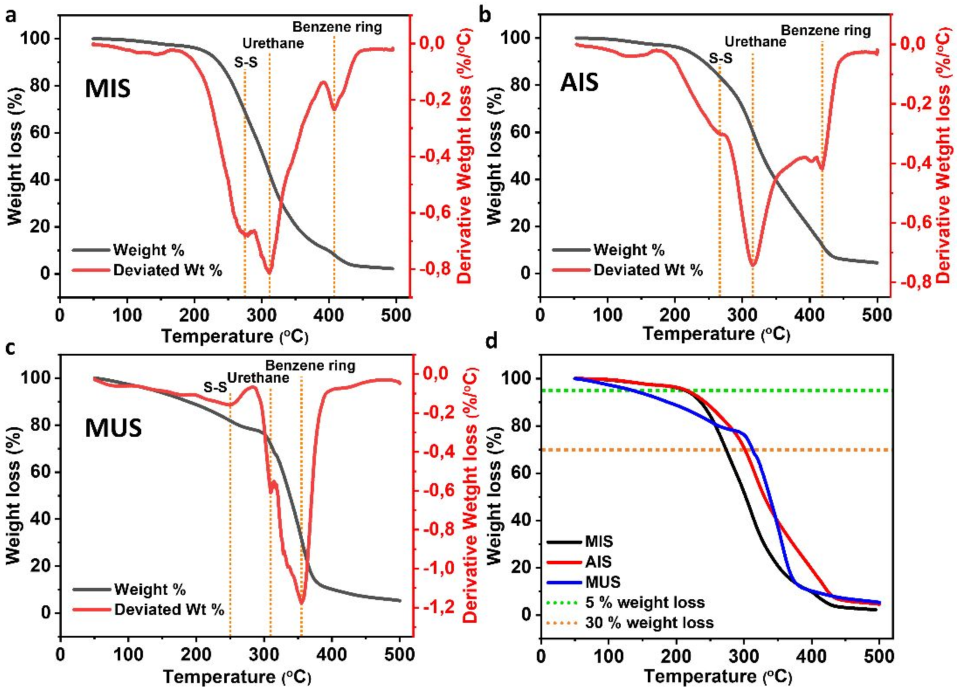

Figure S4. TGA and thermogravimetric-differential curve diagrams of (a) MIS (b) AIS (c) MUS cross-linkers (d) Comparative thermogravimetric analysis of MIS, AIS, and MUS cross-linkers.

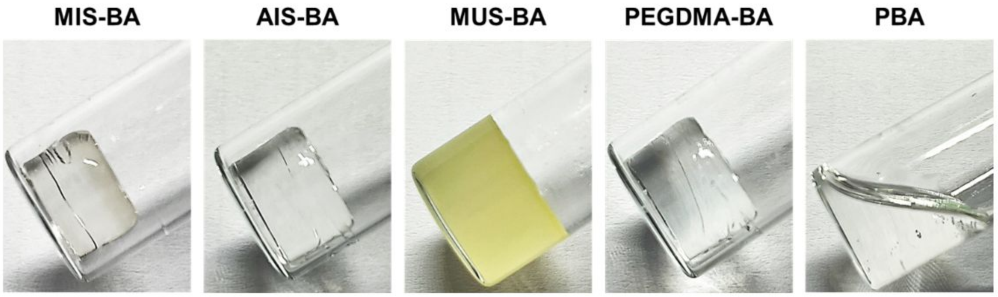

Figure S5. Optical imaging of elastomers and PBA.

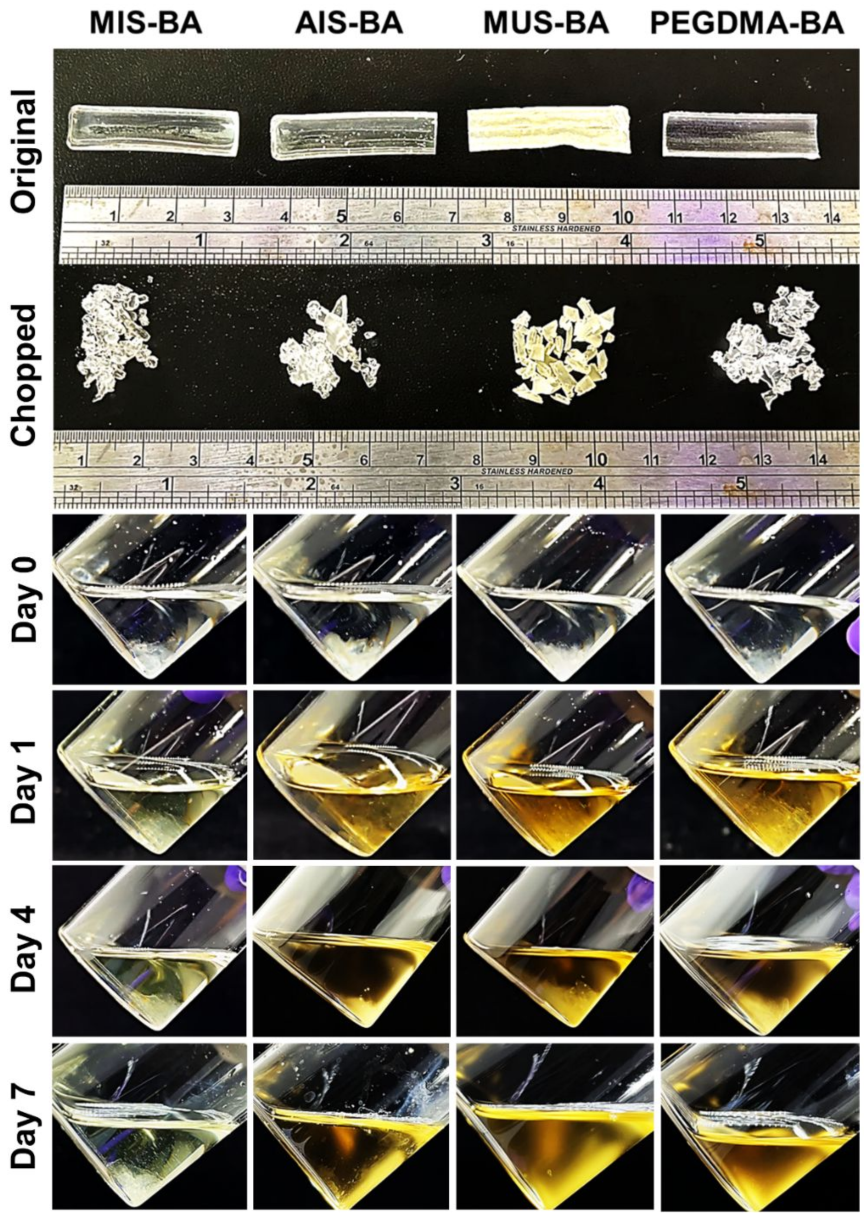

Figure S6. Optical images of the elastomers and the degradation process.

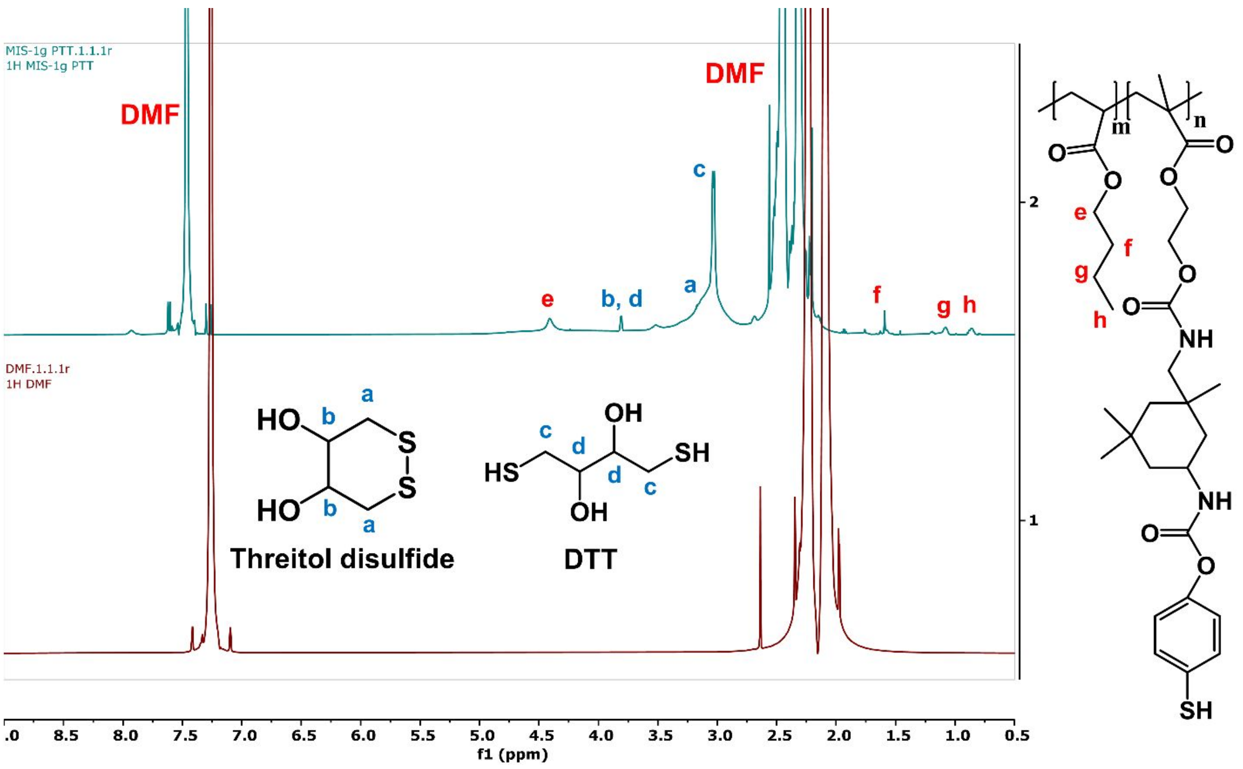

Figure S7. ^{1}H-NMR spectra of degraded MIS-BA and DTT in DMF.

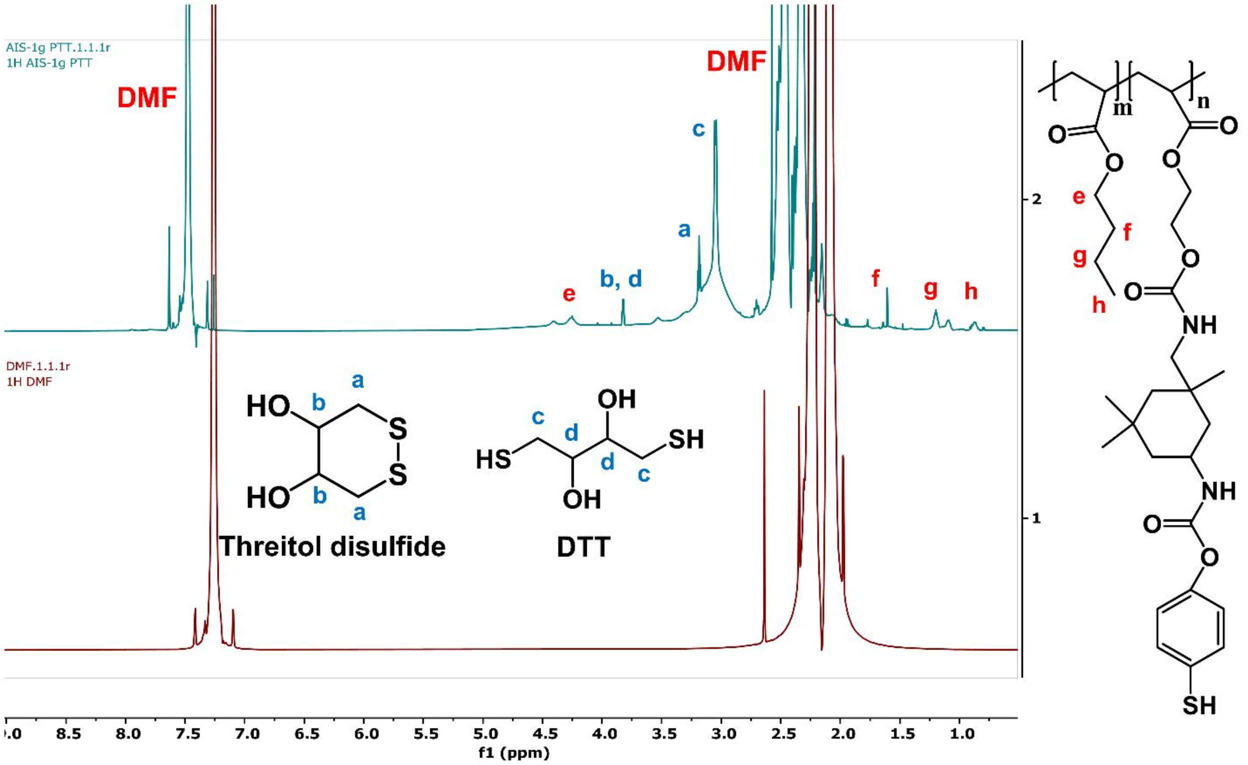

Figure S8. ^{1}H-NMR spectra of degraded AIS-BA and DTT in DMF.

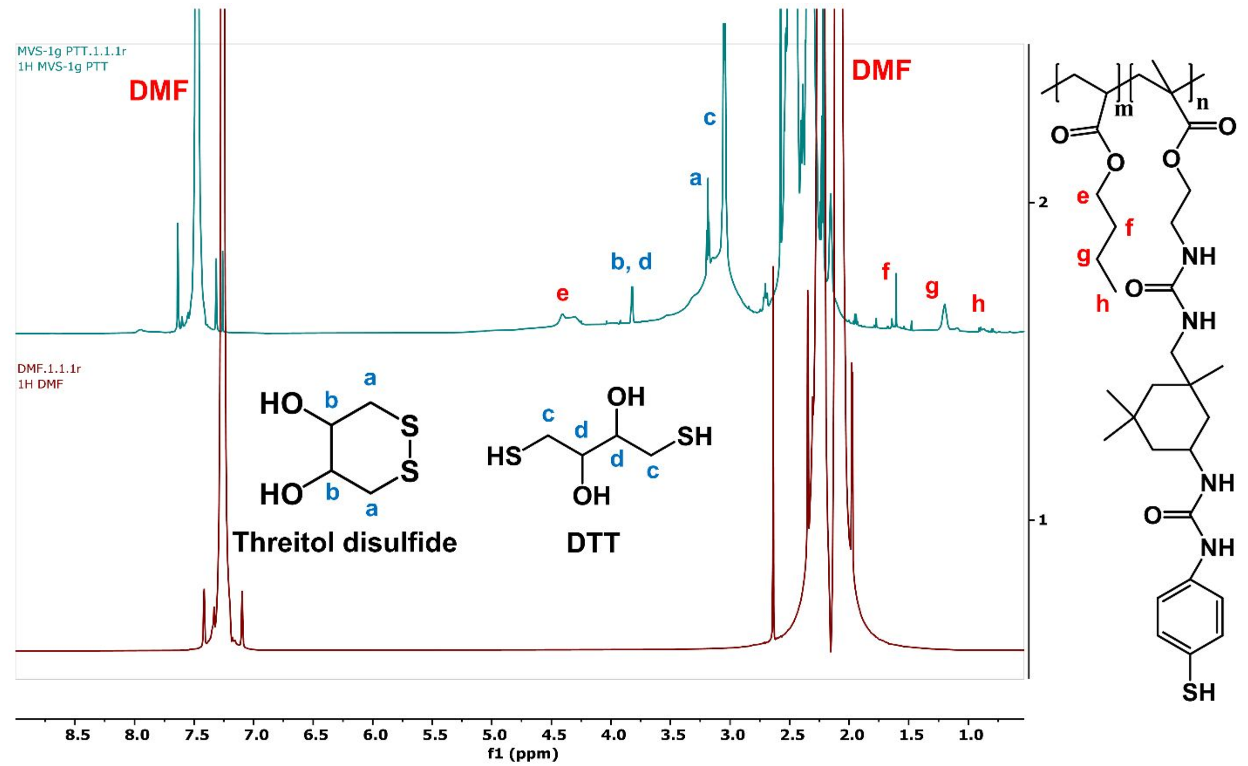

Figure S9. ^{1}H-NMR spectra of degraded MUS-BA and DTT in DMF.
